# Current status of Radiomics for cancer management: Challenges versus opportunities for clinical practice

**DOI:** 10.1002/acm2.12982

**Published:** 2020-07-22

**Authors:** Hua Li, Issam El Naqa, Yi Rong

**Affiliations:** ^1^ Department of Bioengineering University of Illinois at Urbana‐Champaign Urbana IL USA; ^2^ Department of Radiation Oncology Carle Cancer Center, Carle Foundation Hospital Urbana IL USA; ^3^ Department of Radiation Oncology University of Michigan Ann Arbor IL USA; ^4^ Department of Radiation Oncology University of California Davis Cancer Center Sacramento CA USA

## 1 INTRODUCTION

Radiomics, the high‐throughput extraction and analysis of features from medical images, is a promising field for characterizing tumor phenotype and normal tissue injury post‐radiotherapy. Radiomics provides unique opportunities to identify predictive and prognostic imaging biomarkers in noninvasive imaging assays providing so‐called digital biopsies that can be acquired throughout the whole course of cancer treatment. Radiomics have been proved to be associated with underlying gene expression and therapy response, which is an area currently referred to as radiogenomics. Multimodality imaging biomarkers extracted from positron emission tomography (PET), computed tomography (CT), magnetic resonance imaging (MRI), and images in other medical modalities have been shown to have discriminative power for cancer treatment outcome prognosis and prediction. For example, F‐fluoro‐2‐deoxy‐D‐glucose (FDG)‐PET images are the standard of care in tumor quantification of head and neck radiation therapy (RT) and will likely remain so for the foreseeable future. Metabolic tumor volume, defined as the volume of tumor tissues with increase and heterogeneous FDG uptakes, is an important prognostic factor in many malignancies. The radiomics features can complement known first order imaging biomarkers and provide further insights beyond those revealed to naked eyes from medical images.

During the past years, there has been tremendous growth in the radiomics field leading to improved performances in cancer diagnosis, cancer staging, tumor classification, treatment outcome prediction, patient survival, and other clinical practice, compared to other simple clinical biomarkers such as tumor staging, tumor size, human papillomavirus (HPV) status, etc. Clinical applications of radiomics have been widely investigated as well.^1^, ^2^ Radiomics yield great promise to support clinical practice and achieve many promising results. There are many publications and special issues dedicated to the usage of radiomics to support clinical applications in combination with recent spread of advanced machine learning methods.^3^, ^4^ Yet questions remain if the development of radiomics makes it ready for prospective clinical use. Herein, we brought in two medical physics experts both of whom have extensive knowledge in clinical practice and radiomics research. Dr. Hua Li is taking the proposition that “Radiomics poses more challenges than opportunities for clinical practice in cancer management,” whereas Dr. Issam El Naqa argues against it.

Dr. Hua Li is currently a research associate professor in the Department of Bioengineering at University of Illinois at Urbana‐Champaign and a clinical medical physicist at Carle Cancer Center, Carle Foundation Hospital, Urbana, IL. Before joining UIUC and Carle, she was an associate professor in the Department of Radiation Oncology at Washington University in Saint Louis. Dr. Li is certified in Therapeutic, Diagnostic, and Nuclear medical physics by the American Board of Radiology. She has conducted active research in developing advanced machine learning, pattern recognition, and image analysis techniques for applications in radiation therapy and diagnostic imaging. Her current research projects include radiomics‐based prognostic model of cervical cancer habitats, multimodal biomarkers for personalized oropharyngeal cancer treatment, and task‐based image quality assessment and optimization in radiation therapy. Her research projects are funded by the National Institute of Health (NIH).

Dr. EI Naqa worked as a Professor and associate member in Applied Physics and the Michigan institute of data science. He recently accepted the position of founding chair of the department of Machine Learning at Moffitt Cancer Center, Tampa, Fl, where will officially start later this summer. He is a certified Medical Physicist by the American Board of Radiology. He is a recognized authority in the fields of machine learning, data analytics, and oncology outcomes modeling and has published extensively in these areas with more than 180+ peer‐reviewed journal publications and four edited textbooks. He has been a senior member and fellow of several academic and professional societies. His research has been funded by several federal and private grants in Canada and the USA and served on national and international study sections. He acts as a peer‐reviewer and editorial board member for several leading international journals in his areas of expertise.

## 2 OPENING STATEMENTS

### 2.A Hua Li, PhD

The usage of radiomics for reliably and efficiently supporting clinical decision‐making in cancer therapy remains largely immature with many impediments. An effective yet stable methodology model able to select and learn from radiomics (or deep learn from the related images) to support clinical practice of cancer diagnostics, treatment prognosis, and prediction is still desired.^5^, ^6^ In this section, we highlight the main challenges with the hope to guide the design and development of efficient and robust methodology to address these challenges and expedite the clinical applications of radiomics.

#### 2.A.1 Limitations of medical imaging systems

Imaging systems are imperfect and generally acquire indirect measurements of an object property that is affected by multiple sources of noise. Tomographic images reconstructed from such measurements are additionally influenced by the choice of reconstruction method and may contain artifacts. The spatial resolution of imaging systems is limited by the imaging physics and the instrument response. A fundamental question is whether a given imaging system is capable of producing images that contain information that is potentially useful for making predictions related to treatment outcomes or other tasks. For example, high heterogeneity of radiomics exists among images of the same patient but acquired from different imaging systems.^7^ Moreover, the lack of harmonization and standardization for radiomics due to the limitations of imaging systems is an intrinsic issue that needs to be aware of.

#### 2.A.2 Radiomic features yield high redundancy

Conventionally, radiomics is a method in which a large number of features are extracted from images for the purpose of making a statistical inference related to a disease. Given interesting regions (ie, tumor), complementary and quantitative radiomic features, including semantic features (tumor shape, size, locations, etc.), histogram‐based features (mean, median, maximum, entropy, skewness, etc.), textural features, wavelets, and others, can be extracted. However, only a small portion of these extracted features have been proven to be useful. Designing efficient way to determine those informative ones from a large number of extracted features can be a very challenging task.

#### 2.A.3 Radiomic features yield high heterogeneity

Radiomic features, extracted from multimodality images and individual patients, yield high heterogeneity due to varied imaging system principles, numerous imaging protocols and parameters, and intrinsic differences among images from different modalities. The high heterogeneity of radiomics brings additional challenges to determine stable and informative features and properly fuse them to support clinical applications of radiomics.

#### 2.A.4 Radiomic features yield high uncertainty

Uncertainties and variations in delineated tumor shapes and sizes cause high uncertainty of extracted radiomics from images in different imaging modalities and exhibited in individual patients, mainly due to variations among manual, semi‐automatic, or automatic tumor delineation methods.

#### 2.A.5 Uncertainty in clinical outcomes

Due to their partially subjective determination based on physician experience, the Response Evaluation Criteria in Solid Tumor (RECIST) version 1.1, biopsy results, and/or after‐treatment medical images, clinical outcomes yield uncertainty. In another words, partial subjectivity exists and is unavoidable in determining cancer treatment outcomes and other clinical diagnosis and treatment endpoints among patient samples. The outcome uncertainty brings additional challenges of using radiomics for cancer diagnosis and treatment outcome prognosis.

#### 2.A.6 Challenges of small number and imbalanced (skewed) training dataset

Relatively small training patient numbers in comparison to the high‐dimensional radiomic feature space can potentially cause unstable performance of the trained model on unseen cases. In addition, imbalanced (skewed) training patient samples, due to the very different class label rates, significantly affect the model performance as well. Numerous clinical studies demonstrated that the outcome rates for majority cancers are imbalanced. The prediction model trained by unbalanced samples yields high false negative prediction rates on unseen data samples in minority classes. Minority class samples require rebalancing in order to reduce the difficulties in learning them, improve the stability of the selected optimal features, and decrease the predictive error rates on minority classes.

#### 2.A.7 Challenges of using deep learning approaches

Unlike traditional machine learning‐based methods, more and more advanced deep learning‐based radiomics (DLRs) have been adapted to extract deep radiomics for RT applications^8^. In deep learning‐based methods, medical images are directly employed as the input without separating regions‐of‐interest delineation and radiomics extraction from the following classification. The deep learning‐based methods can be treated as a simple end‐to‐end process. For example, CNN or autoencoder architectures, which combine both linear and nonlinear functions, are employed to explore deep features from images and feed to the following deep net for decision‐making. The delineation of tumor or other organs‐of‐interest can be skipped, which reduces the burden to clinicians and the effect of tumor contour uncertainty on the performance of the radiomics‐based applications. However, deep learning networks normally need to be trained with a larger amount of training data in order to meet its learning task and achieve stable performance. Collecting a large amount of proper training dataset in the medical field is a very challenging issue. In addition, selecting and evaluating optimal DL network architectures for clinical applications still requires thoughtful understanding of DL networks considering the intrinsic differences of medical imaging systems compared to other imaging. Therefore, medical image‐specific network design, training, validation, and testing are required for the safe use of DLRs to guide clinical practice.

In summary, imaging system limitations, data redundancy, heterogeneity, uncertainty, and imbalance of the large number of candidate radiomics features, and existing small training sets are the challenges that need to be aware of. A critical barrier hampering the widespread and stable use of imaging biomarkers in clinical practice is the lack of robust tools for identifying prognostic biomarkers from high‐dimensional features that work across patient population. The employment of traditional machine learning and advanced deep learning methods in medical imaging fields also requires some special considerations.

### 2.B Issam El Naqa, PhD

Despite the aforementioned challenges, there is light at the end of the tunnel. Moreover, delaying the adoption of radiomics until all these challenges are resolved is impractical and will miss the current existing opportunities for employing radiomics to support clinical decision‐making. The mere presence of noninvasive nature of medical images and possibility of high spatial and temporal resolution provide major benefits over using simplistic metrics that would overlook the wealth of useful information on tumor’s shape, growth/shrinking over time, heterogeneity that radiomics can provide as discussed below.

#### 2.B.1 New standards and metrology for radiomics are evolving

Many organizations are leading the way to standards for quantitative imaging and its utilization as biomarkers including the RSNA and AAPM, among others. These initiatives and others have led to useful recommendations for repeatability and reproducibility such as the Quantitative Imaging Biomarkers Alliance (QIBA) in MRI and other modalities, for instance.^9^ In addition, consortium such as The Image Biomarker Standardization Initiative (IBSI) specifically designated for standardizing radiomics for high‐throughput image‐based phenotyping.^10^

#### 2.B.2 Multimodality imaging is underutilized

The use of imaging in RT is progressing at a rapid pace. The simplistic use of this existing wealth of imaging modalities is a typical case of information waste that is missing tremendous opportunities that radiomics can bring to bear beyond simple intensity or volumetric metrics that currently dominate the radiological lexicon. This is currently a major deficiency in imaging biomarkers. Radiomics can complement and improve the diagnostic values for systems such as BI‐RADS (breast imaging) or PI‐RADS (prostate imaging). The situation is worse in case of therapeutic studies such as radiation oncology, where such systems are missing and radiomics can fill in this vacuum.

#### 2.B.3 Radiomics can add value to existing clinical or other biomarkers

Current metrics used to support clinical decision‐making fall short of achieving the desired thresholds for fulfilling the clinical need. Hence, the integration or combination with other informative biomarkers can better support the advisable, where radiomics would complement these features and add further value. For example, tissue biopsy is widely employed for detecting and investigating cancerous cells and yielding unreplaceable benefits. However, its reliability is limited by the fact that tumors are spatially and temporally heterogeneous. They cannot capture all the necessary information for an inclusive decision. Additionally, most biopsies are invasive and require very restricted procedure. In addition, biopsies are not viable solution because of the high risk of complications for certain patients. Although biopsy remains the gold standard for cancer diagnosis, combining it with radiomics can better capture hidden intratumoral heterogeneity. Radiomics may be used to facilitate biopsies by detecting more suspicious locations, and can provide complementary information for cancer diagnosis, outcome prognosis, and prediction. In addition, the integration of radiomics and other biomarkers, such as genomics, clinical, and demographic biomarker, and electronic health record (EHR), can bring more opportunities and promising applications in supporting RT clinical practice. Multimodal biomarkers including radiomics can better support clinical practice.

#### 2.B.4 Advances in data science are benefiting radiomics

Though radiomics have its own issues like redundancy, uncertainty, and instability like any other type of ‘omics (genomics, transcriptomics, and proteomics), which did not limit their utilization and should not limit radiomics as well. Indeed, dealing with large number of variables is major challenge in statistical modeling and machine learning. However, advances in machine learning and deep learning specifically opened the door for complementing feature‐based methods with featureless (machine learnt) methods that are rapidly advancing and would make the future of radiomics even brighter.

In short, radiomics has its own share of challenges but these should not be a hindrance to its cautious use, given the facts that radiomics benefits far outweigh its risks and the missed opportunities that it can offer.

## 3 CONCLUSION/AGREEMENT

### 3.A Hua Li, PhD and Issam El Naqa, PhD

In spite of those abovementioned challenges, there are promising opportunities of continuously employing radiomics to support clinical decision‐making considering the unique image characteristics such as the noninvasive nature of medical images, the high spatial and temporal resolution, and the promising development of advanced imaging techniques. For example, tissue biopsy cannot capture all the necessary information (ie, spatial and temporal heterogeneity) of the tumor characteristics for an inclusive decision although it is widely employed for detecting and investigating cancerous cells. Radiomics can facilitate biopsies by detecting more suspicious locations by providing complementary information for cancer diagnosis, outcome prognosis, and prediction. The integration or combination of radiomics with other informative biomarkers can better support the clinical decision‐making.

Though the path is promising there may be some bumps along the way to fulfill the full promise of radiomics in clinical practice including accounting for intrinsic imaging properties and recognition of machine/deep learning technique limitations. The example of supplementing clinical biopsies with a digital one is a low hanging fruit case that radiomics can be effectively demonstrated. Other promising areas include clinical decision support systems with other ‘omics, which are evolving at a rapid pace and prospective clinical trial design, which is yet to be developed and can be of great potential.^11^

Being aware of the intrinsic properties of imaging systems and radiomics, the advantages and disadvantages of the traditional machine learning techniques and advanced deep learning techniques, it is expected that the aforementioned promising opportunities will lead to more applications of radiomics to support clinical practice in RT and improve its outcomes. Integrating information carried by radiomics into personalized treatment is likely to keep growing given the increased role of images in medical practice. Imaging biomarkers will keep playing a critical role in supporting personalized cancer diagnosis and treatment in the near future and radiomics will be an indispensable tool to make best use of these images.
